# Hepatic sinusoidal obstruction syndrome and the search for a modifiable endothelial state

**DOI:** 10.1002/imt2.70190

**Published:** 2026-11-11

**Authors:** Ziming Zhao, Zijun Zhang, Fengling Liu, Haolu Wang, Di Yu, Chuanrui Xu, Xin Chen, Yang Xiao, Qing Yu, Qianrong Xiong, Eric C.H. Lai, Qingyi Tong, Xiaoping Chen, Yonghui Zhang

**Affiliations:** ^1^ Hubei Key Laboratory of Natural Medicinal Chemistry and Resource Evaluation, School of Pharmacy, Tongji Medical College Huazhong University of Science and Technology Wuhan China; ^2^ Department of Pharmacy, Maternal and Child Health Hospital of Hubei Province, Tongji Medical College Huazhong University of Science and Technology Wuhan China; ^3^ Internal Medicine Services, The Prince Charles Hospital Chermside Australia; ^4^ Frazer Institute, Faculty of Health, Medicine and Behavioural Sciences The University of Queensland Woolloongabba Australia; ^5^ University of Hawaii Cancer Center Honolulu Hawaii USA; ^6^ Training Centre for Healthcare Management & Clinical Technology, Pamela Youde Nethersole Eastern Hospital Hong Kong China; ^7^ Hepatic Surgery Center, Institute of Hepatobiliary‐Pancreatic Surgery, Tongji Hospital, Tongji Medical College Huazhong University of Science and Technology Wuhan China

## Abstract

Pyrrolizidine alkaloid (PA) exposure, haematopoietic stem cell transplantation (HSCT), and repeated oxaliplatin treatment provide distinct contexts for sinusoidal endothelial injury, with differing mechanisms and courses. Oxaliplatin‐associated abnormalities may resolve or persist. Illustrated features are conceptual, not validated clinical stages or a shared temporal sequence. Recognition varies by setting and age; the proposed gap remains unvalidated. Current biomarkers and imaging do not establish the reversibility of liver sinusoidal endothelial cell (LSEC) injury. Clinical care remains etiology‐specific. Testing whether an endothelial profile can guide treatment selection requires endothelial‐associated measures, independent vascular assessment, and prospective evidence of differential treatment benefit. No endothelial‐state‐based treatment threshold has been validated.

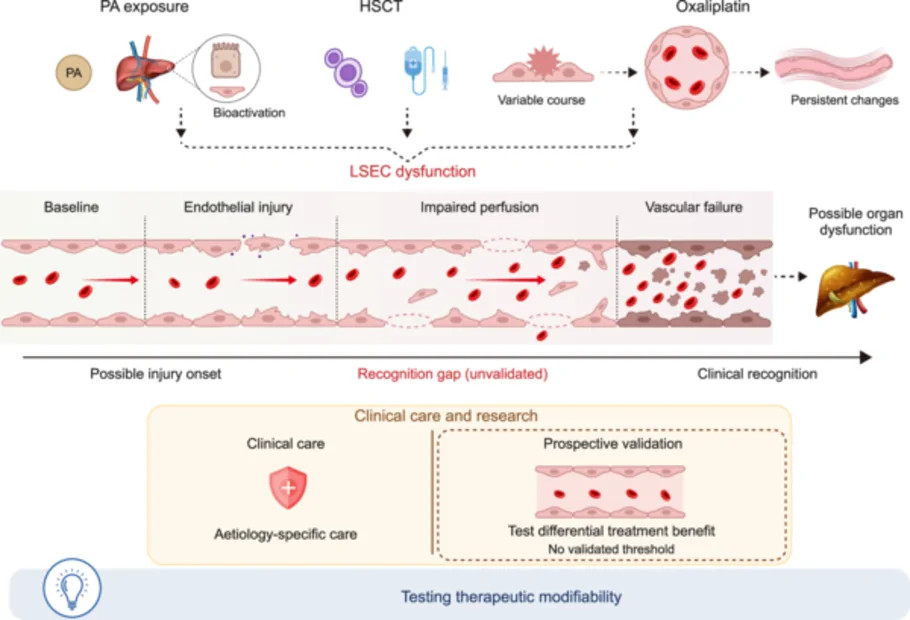

## CONFLICT OF INTEREST STATEMENT

The authors declare no conflicts of interest.

## ETHICS STATEMENT

No animals or humans were involved in this study.

## AI STATEMENT

During the preparation and revision of this work, the authors used DeepSeek to assist with language refinement, manuscript organization, and drafting. All outputs were critically reviewed and revised by the authors, who take full responsibility for the content of the manuscript. No generative AI tool was used to generate or modify scientific data or final figures.

To the Editor,

Hepatic sinusoidal obstruction syndrome (HSOS), historically termed hepatic veno‐occlusive disease (VOD), is characterized by sinusoidal endothelial injury and impaired hepatic perfusion [1, 2]. Following haematopoietic stem cell transplantation (HSCT), it is termed HSCT‐associated VOD/SOS. Disease caused by pyrrolizidine alkaloid (PA) exposure is termed PA‐HSOS. Repeated oxaliplatin treatment can also cause sinusoidal injury without overt clinical manifestations [1, 2, 3, 4]. These conditions all involve liver sinusoidal endothelial cell (LSEC) injury, but their causes, clinical courses, and treatment approaches differ.

The European Society for Blood and Marrow Transplantation (EBMT) distinguishes probable, clinical, and proven VOD/SOS in adults [5]. The 2026 European Association for the Study of the Liver (EASL) guidelines require histological confirmation for proven disease; an elevated hepatic venous pressure gradient alone is insufficient [1]. For PA‐HSOS, the Nanjing criteria combine a history of PA exposure with compatible clinical and imaging findings [2, 6]. These approaches support diagnosis but do not directly establish whether treatment can limit further endothelial injury or improve sinusoidal blood flow. Experimental studies suggest that endothelial injury can precede overt disease [7, 8]. Whether this interval can be identified in patients, and how long it lasts, remain uncertain. Studies in each etiological setting are needed to determine whether treatment during this interval improves outcomes. We therefore outline how future studies could test whether markers of endothelial injury and measurements of sinusoidal vascular function identify patients more likely to benefit from treatment targeting the injured endothelium. This hypothesis requires prospective testing in intervention studies.

## REVIEW APPROACH

This focused narrative synthesis used targeted, non‐exhaustive searches of PubMed‐indexed records and primary publisher materials, updated to 14 October 2026. Searches combined “sinusoidal obstruction syndrome” or “veno‐occlusive disease” with PA exposure, HSCT, oxaliplatin, LSECs, biomarkers, prevention, and treatment; guideline reference lists were also examined. Selected publications from 2000 to 2026 addressed etiology, diagnosis, endothelial injury, or management. Accessible full texts were assessed qualitatively for design, setting, timing, and limitations, distinguishing patient observations from human‐derived models and animal experiments. No formal risk‐of‐bias scoring or quantitative pooling was performed.

## ETIOLOGY‐SPECIFIC MECHANISMS AND CLINICAL COURSES

PA‐HSOS follows hepatic bioactivation of toxic plant alkaloids [2]. In an 86‐case validation cohort, biopsies documented zone‐3 endothelial swelling, necrosis, and detachment, with sinusoidal dilatation and congestion [6]. Blood pyrrole–protein adducts support exposure attribution but do not establish endothelial reversibility [2, 6]. Recent work combining observations from 30 patients with human LSEC and animal experiments links hemolysis and impaired hemoglobin clearance to LSEC injury [9]. A chronic PA mouse model implicates plasminogen activator inhibitor‐1 (PAI‐1)–p53 signaling in endothelial senescence and junctional injury. The same study reported elevated serum PAI‐1 in 16 patients; causal and therapeutic evidence remains predominantly experimental [10].

HSCT‐associated VOD/SOS arises amid conditioning toxicity, prior hepatotoxic exposure, inflammation, altered permeability, and hemostatic disturbance [5]. These processes may overlap rather than follow a uniform sequence. Circulating abnormalities may also reflect other transplant complications rather than isolated hepatic endothelial injury [11].

Oxaliplatin‐associated sinusoidal injury can occur without overt clinical manifestations. A study examined resection specimens from 22 patients treated with fluorouracil, leucovorin, and oxaliplatin (FOLFOX) and 18 untreated patients, alongside a corresponding porcine model. Treated groups exhibited LSEC morphological changes and matrix metalloproteinase‐9 upregulation, without hepatomegaly, ascites, or coagulative hepatocyte necrosis [3]. In a separate longitudinal cohort, most imaging abnormalities resolved, although chronic portal hypertension persisted in five of 356 patients [4]. Etiology‐specific clinical profiles and study details are provided in Tables S1 and S2.

## A HYPOTHESIS‐GENERATING FRAMEWORK OF LSEC DYSFUNCTION

Five potentially overlapping domains organize the evidence: redox–metabolic disturbance, structural and junctional alterations, barrier disruption, inflammatory–hemostatic activation, and microcirculatory dysfunction (Figure 1 and Table S2). These domains are neither validated clinical states nor successive stages; a common temporal sequence and shared recovery window have not been established in humans.

**Figure 1:**
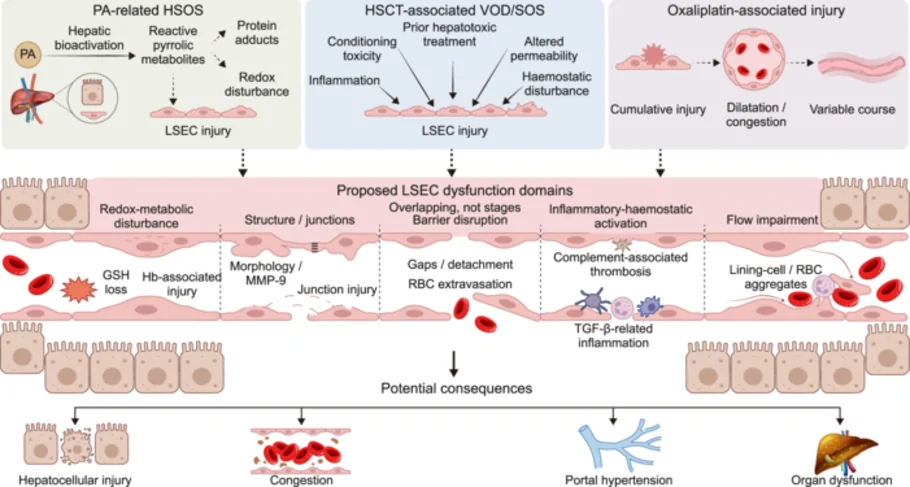
A hypothesis‐generating framework for sinusoidal endothelial dysfunction. Etiology‐specific insults are shown alongside five potentially overlapping domains of LSEC dysfunction. The PA pathway integrates patient observations with experimental mechanisms, including hepatic bioactivation to reactive pyrrolic metabolites, protein adduct formation, and redox disturbance. The HSCT pathway synthesizes clinical guidance, whereas the oxaliplatin pathway draws on human tissue observations, imaging findings, and porcine experiments. Redox‐metabolic evidence includes GSH‐related findings in MCT‐treated rats and Hb‐associated LSEC injury in PA experiments. Structural findings include oxaliplatin‐associated LSEC morphological changes and MMP‐9 upregulation in human tissue and a porcine model, together with junctional injury in PA mouse and cell experiments. Time‐resolved MCT rat experiments link endothelial gaps, RBC extravasation, and lining‐cell detachment to lining‐cell/RBC aggregates and impaired flow. Complement‐associated thrombosis and TGF‐β‐related inflammation are illustrated using an ATG exposure model involving vascularised liver organoids derived from human induced pluripotent stem cells and engrafted in mice. Histological corroboration for this model was limited to one liver‐transplant case; applicability to HSCT recipients remains uncertain. The five domains are neither validated clinical states nor successive stages. Dashed arrows connecting aetiologies to the framework denote proposed cross‐setting links, whereas dashed links in the oxaliplatin pathway indicate a variable course. A shared human temporal sequence and recovery window remain unvalidated. Altered sinusoidal structure and perfusion may contribute to congestion, portal hypertension, hepatocellular injury, and organ dysfunction, with etiology‐specific clinical courses. ATG, antithymocyte globulin; GSH, glutathione; Hb, hemoglobin; HSCT, haematopoietic stem cell transplantation; HSOS, hepatic sinusoidal obstruction syndrome; LSEC, liver sinusoidal endothelial cell; MCT, monocrotaline; MMP‐9, matrix metalloproteinase‐9; PA, pyrrolizidine alkaloid; RBC, red blood cell; TGF‐β, transforming growth factor‐β; VOD/SOS, veno‐occlusive disease/sinusoidal obstruction syndrome.

In monocrotaline (MCT)‐treated rats, continuous intraportal glutathione (GSH) infusion preserved endothelial GSH and prevented SOS when started before exposure; initiation 24 h after MCT was partially protective. Withdrawal on Day 5 was followed by severe disease on Day 6 [7]. These experiments demonstrate protection with continued supplementation, not reversal of established human HSOS. Hemolysis‐derived hemoglobin also promotes LSEC activation and ferroptotic injury in PA experiments [9].

Structural alterations include the oxaliplatin‐associated changes and PA‐associated junctional injury described above [3, 10]. In time‐resolved MCT experiments, endothelial gaps preceded erythrocyte extravasation and dissection of the sinusoidal lining; detached lining cells, erythrocytes, and adherent monocytes formed aggregates that obstructed flow [8]. Physiological fenestrae should not be equated with pathological gaps or denudation. These observations connect barrier disruption to microcirculatory dysfunction in that model, without establishing equivalent temporal relationships across human aetiologies.

An antithymocyte globulin (ATG) exposure model used vascularised liver organoids derived from human induced pluripotent stem cells and engrafted in mice. Complement‐associated thrombosis preceded transforming growth factor‐β‐related inflammatory activation [12]. A single ATG‐associated SOS case after liver transplantation provided limited clinical corroboration; applicability to HSCT recipients remains uncertain [12].

Therapeutic modifiability can involve preserving function or limiting progression without restoring vascular architecture. Serial Doppler imaging and liver stiffness measurements track vascular consequences but are influenced by haemodynamics and tissue changes [1, 13]. Histological improvement in selected PA‐HSOS patients does not define an early LSEC‐specific recovery window [14].

## FROM RECOGNITION SIGNALS TO TREATMENT TIMING

Exposure attribution, risk prediction, diagnosis, and severity assessment should be distinguished from therapeutic modifiability (Figure 2). Current biomarkers and imaging do not establish the reversibility of LSEC injury or define a validated threshold for treatment selection based on endothelial state. In a prospective study of 80 pediatric haematopoietic cell transplantation recipients with risk factors, 10 developed SOS. A Day‐3 panel comprising l‐ficolin, hyaluronic acid, and soluble ST2, a receptor for interleukin‐33, yielded 80% sensitivity, 73% specificity, and 29.6% positive predictive value [11]. Few events, modified Seattle criteria, and the pediatric setting limit generalization; predictive performance does not establish treatment benefit. In ELASTOVOD, serial liver stiffness in 941 HSCT recipients was associated with disease presence and severity and declined during recovery, without demonstrating endothelial repair or a pre‐emptive treatment threshold [13].

**Figure 2:**
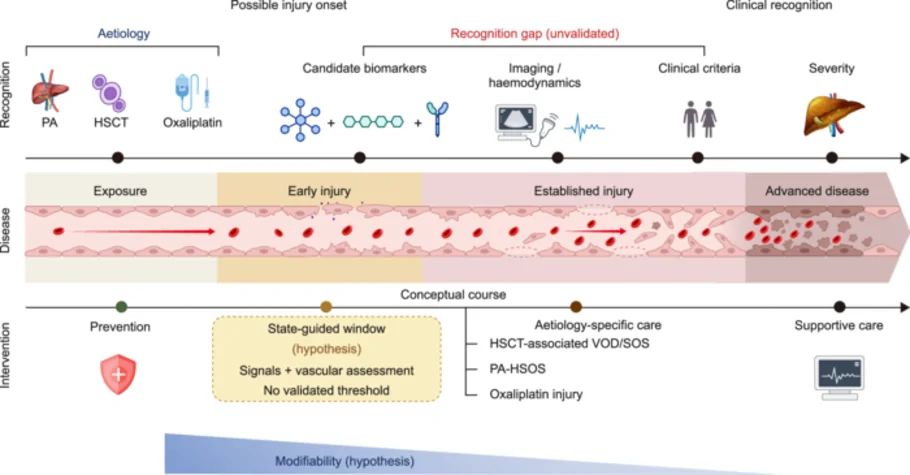
Clinical recognition and intervention opportunities in HSOS and related sinusoidal injury. Exposure attribution, risk prediction, diagnosis, and severity assessment address different clinical questions. Candidate risk biomarkers include l‐ficolin, HA, and sST2 evaluated in a pediatric HCT cohort. Imaging and haemodynamic measures support diagnosis and severity assessment, while clinical criteria and organ dysfunction inform recognition and severity classification. The illustrated course and recognition gap are conceptual and do not establish a validated shared timeline; recognition patterns vary by etiology and age. Current biomarkers and imaging do not establish the reversibility of LSEC injury. Established prevention and treatment are distinguished from the proposed endothelial‐state‐guided window. Prevention includes UDCA prophylaxis in HSCT. Clinical management includes defibrotide for HSCT‐associated VOD/SOS according to age and severity; exposure cessation and supportive care for PA‐HSOS, with anticoagulation in selected patients after assessment of bleeding risk and contraindications; and stopping oxaliplatin where feasible while individualizing oncology decisions for oxaliplatin‐associated SOS. Supportive care may be required throughout the course. Evaluating endothelial profiles for treatment selection within the proposed window requires endothelial‐associated measures, independent vascular assessment, and prospective evidence of differential treatment benefit. No endothelial‐state‐based treatment threshold has been validated. The modifiability gradient represents a hypothesis rather than a validated temporal scale, and established injury should not be assumed to be uniformly irreversible. HA, hyaluronic acid; HCT, haematopoietic cell transplantation; sST2, soluble interleukin‐33 receptor ST2; UDCA, ursodeoxycholic acid.

Prevention of HSCT‐associated VOD/SOS includes risk reduction and ursodeoxycholic acid [1]. The randomized HARMONY trial did not demonstrate a prophylactic benefit of defibrotide in 372 high‐ or very‐high‐risk adult and pediatric recipients: day‐30 SOS‐free survival was 67% with defibrotide plus standard care versus 73% with standard care alone [15]. EASL generally advises against prophylactic defibrotide, whereas the 2026 EBMT consensus supports risk‐adapted use in selected high‐risk children [1, 16]. Prevention, treatment, and experimental biomarker‐guided pre‐emption should remain distinct.

In adults, mild HSCT‐associated VOD/SOS requires supportive care and close reassessment; defibrotide is recommended for severe disease and may be considered for moderate disease [1, 5]. EASL recommends initiating defibrotide as soon as SOS is clinically suspected in children, regardless of severity [1]. A study comparing 102 treated patients with severe disease and multiorgan failure against 32 historical controls reported day‐100 survival of 38.2% versus 25.0%, respectively [17]. Earlier post‐diagnosis initiation was associated with better day‐100 survival among 573 treated recipients, but timing was not randomized [18]. Both comparisons remain vulnerable to confounding. These findings do not establish reversal of a defined LSEC state or efficacy in other aetiologies. Transjugular intrahepatic portosystemic shunting (TIPS) may be considered in selected patients with severe or very severe HSCT‐associated disease who deteriorate rapidly despite medical treatment, including defibrotide [1]. These indications cannot be inferred from isolated experimental biomarker abnormalities.

PA‐HSOS prevention emphasizes avoiding toxic PA‐containing plants. Management begins with exposure cessation and supportive care [2]. Chinese expert consensus recommends early anticoagulation for acute or subacute PA‐HSOS after excluding contraindications, including severe hemorrhage or a bleeding tendency [2]. Suggested regimens use low‐molecular‐weight heparin combined with or followed by warfarin, with renal function assessment and anticoagulant monitoring [2]. In a 249‐patient retrospective cohort, anticoagulation was associated with improved remission and survival, particularly in selected patients with mild or moderate disease [19]. A 49‐patient series reported bleeding in 6/30 anticoagulated patients versus 1/19 controls, without a statistically significant difference (*p* = 0.15) [20]. Treatment selection, heterogeneous regimens, and concomitant TIPS limit causal and safety inference. TIPS may be considered after inadequate medical response for refractory ascites or severe portal‐hypertensive complications [2, 14]. In a 164‐patient retrospective cohort, six of seven patients with paired biopsies showed histological improvement; selection and absent paired non‐TIPS controls prevent attribution to TIPS alone [14].

For oxaliplatin‐associated SOS, EASL recommends histological confirmation and identifies no approved pharmacological prevention or treatment. Management includes stopping the drug where feasible and individualizing chemotherapy and surgical decisions; available evidence is insufficient to recommend for or against defibrotide [1]. TIPS may be considered for severe portal‐hypertensive complications in selected patients, although supporting evidence is limited [1]. Further study details and limitations are provided in Tables S1 and S2.

## DEFINING AND TESTING AN ENDOTHELIAL PROFILE FOR RESEARCH

An endothelial injury profile proposed for research should require an identified aetiology, a change exceeding measurement variability in at least one prespecified endothelial‐associated measure on repeated assessment, and concordant findings from an independent assessment of hepatic vascular function. Blood panels, liver stiffness, and Doppler measurements capture associated stress or vascular consequences, not LSEC integrity [11, 13]. Signals, directions of change, sampling intervals, and concordance rules should be derived within each aetiological setting and fixed before external validation. For pretreatment stratification, only measurements obtained before the intervention being evaluated should be used. Neither this profile nor preserved perfusion establishes reversibility or a treatment threshold. Establishing the profile's value for treatment selection requires prospective evidence that treatment benefit differs according to the prespecified profile.

Longitudinal assessment should begin before HSCT conditioning, include assessments before, during, and after oxaliplatin treatment, and start at the earliest PA‐HSOS assessment. PA‐HSOS cohorts enrolled after clinical onset cannot alone establish a preclinical recognition window. Serial signals, vascular assessments, and outcomes should be interpreted alongside infection, volume status, and other confounders. Clinically indicated tissue sampling may support validation.

Experiments must distinguish post‐injury treatment from prevention: the experimental PAI‐1 inhibitor TM5441 was administered after 28 days of PA exposure [10], whereas recombinant human thrombomodulin preceded FOLFOX in pigs [3]. Exposure duration alone does not establish pretreatment endothelial injury. Human LSEC or multicellular flow experiments should document injury before treatment and assess endothelial continuity, size‐appropriate permeability, blood‐cell adhesion, and perfusion. Physiological fenestrae and pathological loss of continuity require distinct functional interpretation.

Prospective studies should test a prespecified profile‐by‐treatment interaction. Where clinical equipoise permits, randomization within prespecified profile strata should compare a defined intervention plus etiology‐specific standard care with standard care alone; indicated care must be maintained in all groups without delay. Outcomes should reflect etiology: severe disease, multiorgan dysfunction, and day‐100 survival after HSCT; persistent portal‐hypertensive complications after PA exposure; and hepatic dysfunction or surgical morbidity after oxaliplatin. Analyses should address baseline severity, competing outcomes, and time‐dependent treatment selection. Neither earlier detection of biomarker changes nor a decline in biomarker levels alone establishes treatment benefit.

Sinusoidal endothelial injury links these settings, but mechanisms and clinical courses differ. Earlier recognition should support guideline‐based, etiology‐specific care. The framework organizes hypotheses rather than clinical stages or treatment thresholds. Aetiology‐specific cohorts, post‐injury experiments, and prospective intervention studies are needed to determine whether treatment benefit differs between patients with different endothelial profiles.

## AUTHOR CONTRIBUTIONS

**Ziming Zhao**: Visualization; writing—original draft; writing—review and editing. **Zijun Zhang**: Writing—original draft; writing—review and editing. **Fengling Liu**: Investigation; writing—review and editing. **Haolu Wang**: Writing—review and editing. **Di Yu**: Writing—review and editing. **Chuanrui Xu**: Writing—review and editing. **Xin Chen**: Writing—review and editing. **Yang Xiao**: Investigation; writing—review and editing. **Qing Yu**: Writing—review and editing; investigation. **Qianrong Xiong**: Investigation; writing—review and editing. **Eric C.H. Lai**: Writing—review and editing. **Qingyi Tong**: Conceptualization; supervision; writing—review and editing. **Xiaoping Chen**: Conceptualization; supervision; writing—review and editing. **Yonghui Zhang**: Conceptualization; supervision; writing—review and editing. **Ziming Zhao and Zijun Zhang:** contributed equally to this work. All authors have read the final manuscript and approved it for publication.

## Supporting information


**Table S1:** Aetiology‐specific clinical profiles of HSOS and related sinusoidal injury. **Table S2:** Principal clinical and experimental studies informing the endothelial framework.

## Data Availability

No new data were generated or analyzed in this article. Supplementary materials (tables, graphical abstract, slides, Chinese translated version, and updated materials) may be found at the online DOI or iMeta Science http://www.imeta.science/.
